# Long-Term Oxygen Therapy in COPD: Factors Affecting and Ways of Improving Patient Compliance

**DOI:** 10.1155/2011/325362

**Published:** 2011-09-15

**Authors:** Stamatis Katsenos, Stavros H. Constantopoulos

**Affiliations:** ^1^Department of Pneumonology, University Hospital of Ioannina, 45110 Ioannina, Greece; ^2^Department of Pneumonology, Medical School, University of Ioannina, 45110 Ioannina, Greece

## Abstract

Long-term oxygen therapy (LTOT) is the cornerstone mode of treatment in patients with severe chronic obstructive pulmonary disease (COPD) associated with resting hypoxaemia. When appropriately prescribed and correctly used, LTOT has clearly been shown to improve survival in hypoxemic COPD patients. Adherence to LTOT ranges from 45% to 70% and utilization for more than 15 hours per day is widely accepted as efficacious. Although several studies have addressed the level of patients' adherence to LTOT, few have suggested or evaluated interventions that conduce to compliance enhancement. The lack of sufficient data regarding COPD patients following oxygen prescription is an enormous void that must be duly confronted to augment clinical effectiveness and cost containment for the long term use. The present review article highlights factors influencing the compliance of patients using LTOT and emphasizes novel strategies and interventions that may prove to be of significant benefit given the remarkably little current research appraising this issue. Therefore, additional research should be promptly performed to verify the efficacy of newly designed approaches in improving the outcomes of patients receiving LTOT.

## 1. Introduction

It is well established that long-term oxygen therapy (LTOT) is the only therapeutic modality proven to alter the late course of chronic obstructive pulmonary disease (COPD). Particularly, two landmark studies, the Nocturnal Oxygen Therapy Trial (NOTT) and the British Medical Research Council (MRC) conducted in the late 1970s have explicitly demonstrated that LTOT (when used for more than 15 hours/day) improves survival rates in patients with severe COPD associated with resting hypoxemia [1, 2]. In terms of maximum benefit, continuous oxygen administration (≥15 h/d) is superior to intermittent or nocturnal use [3]. There is also accumulating evidence that LTOT has favourable effects on other outcome measures, including depression, cognitive function, quality of life, exercise capability, and frequency of hospitalization [4–10]. Moreover, it stabilizes and sometimes reverses the progression of pulmonary arterial hypertension and it diminishes as well cardiac arrhythmias and electrocardiographic findings indicative of myocardial ischemia [11, 12].

The effectiveness of LTOT in improving survival has been substantiated only in stable COPD patients with severe chronic hypoxemia (PaO_2_ less than 55 mmHg (7.3 kPa) or PaO_2_ ranging from 56 to 59 mmHg (7.4–7.8 kPa) in presence of signs of cor pulmonale, hematocrit > 55%) [13]. The resultant clinical benefits of LTOT are contingent on the treatment compliance, the treatment duration, and the adequate correction of hypoxemia. 

Despite the generally recommended daily duration of oxygen use (>15 h/day) to achieve its goals, the adherence to LTOT seems to be poor according to the existing literature [14]. Furthermore, this therapy incurs great expenses to the healthcare systems worldwide because of several hundred of thousands of COPD patients receiving supplemental oxygen and the high expenditures pertinent to durable delivery oxygen equipment. In particular, it is estimated that 1 million patients receive LTOT in the USA with total Medicare reimbursements for costs related to O_2_ exceeding $ 2 billion/year [15, 16]. It is likely that a great deal of money is dissipated since several studies report inadequate adherence rates to this treatment [17].

The aim of this paper is to review nonadherence to LTOT for COPD within the context of chronic care management, factors affecting suboptimal compliance and methods evaluating it as well as strategies that may enhance it. 

## 2. LTOT Prescription

The actual current guidelines are in strong agreement in recommending LTOT for severely hypoxaemic COPD patients (PaO_2 _< 55 mmHg, < 7.3 kPa), whereas some differences have been observed in patients with moderate hypoxaemia (55 < PaO_2 _< 60 mmHg, 7.4 < PaO_2_ > 8 kPa) regarding the criteria which should be associated with PaO_2_ values [18].

Much of the initial research for LTOT addressed the precision of the prescription. In particular, the prescription of oxygen administration for at least 15 h/day is considered to be adequate and it represents one variable that is associated with effective usage [19]. Howard et al. reported that physicians “varied widely in their prescribing habits”. 36% out of LTOT patients were prescribed less than 15 hours per day thus reducing optimal dosage [20]. Walshaw and coworkers concluded that an effective prescription and compliance was associated with a respiratory physician more often than a family doctor [21]. Granados et al. mentioned that 58% of the selected sample met the criteria for oxygen therapy, of these 80.5% (29/36) were correctly prescribed with corrected hypoxemia [22]. Another study found that 55% of patients had not received thorough written instructions regarding the use of LTOT by their physician and 63% were not aware of LTOT importance in the therapeutic management of their disease [23]. The lack of explicit prescription instructions and prescription review does limit patient adherence. 

## 3. LTOT Adherence

The minimum recommended duration of LTOT is 15 h/day thus representing an adequate oxygen adherence, as it has been established and defined by international guidelines on domiciliary LTOT. Several studies have evaluated compliance to LTOT showing rates ranging from 45 to 70% [20–32]. These clinical trials have determined the extent of patients' oxygen use as well as identified problems. Annotations from these studies indicate potential research direction such as patient and oxygen provider education and/or postprescription support.  Table 1 summarizes these studies with their associated discussion and suggested research. The inadequate adherence to LTOT further suggests that patients may not strongly perceive the clinical benefits that have been described by its continuous use. Furthermore, suboptimal adherence has been reported as an independent modifiable risk factor of frequent COPD exacerbations necessitating hospital admissions thus increasing health care costs [33].

## 4. Risk Factors for LTOT Noncompliance

A multitude of factors has been proposed and identified having negative impact on patients' compliance with LTOT. In particular, Cullen attempted to classify all the possible implicated factors in poor adherence to oxygen therapy, as shown in Table 2 [14]. 

Several studies have thoroughly reported the related reasons to a patient's reluctance to wear a cannula or receive oxygen source device for at least 15 h/day. In the first conducted study in early 1980s, only two of 14 patients receiving oxygen through concentrator were complied with the prescribed dose of 15 h/day. The nonadherence was attributed to prescribed flow rate, machine problems, and hypercapnia [25].

Oxygen therapy has induced restriction as well as it has turned out to be threatening to mastery over symptoms because of concerns about dependency and addiction [34]. Patients receiving oxygen from a stationary source are tethered to it, and this in itself may limit their activities of daily living. Many ambulatory sources are bulky and heavy, discouraging, if not preventing, ambulation by elderly patients and others with impaired strength and endurance [35]. 

Patients may also experience side effects due to oxygen use such as nasal dryness, nasal bleeding, dizziness, some loss of taste and smell, and unpleasant cosmetic effects. These are more noticeable when the oxygen is delivered through dual prong nasal cannulae. Kampelmacher et al. in their circumstantial study investigated the complaints of patients prescribed LTOT. Of 528 participants, 108 were smokers and 15 of these smoked while using oxygen. 20% of patients used oxygen less than prescribed due to treatment difficulties, lack of breathlessness, or fear of oxygen addiction. Nonadhering patients were primarily males and were ashamed of their therapy and the associated stigma. Patient complaints were related to restricted autonomy (50%), the delivery device (41%), oxygen source (38%), feeling ashamed (38%), and treatment duration (8%) [36]. 

Many patients may limit ambulatory O_2_ administration because of embarrassment, not wanting to be seen publicly with the recognizable stigma of smoking-related lung disease. Oliver studied COPD patients' views of their impaired lungs *via* semistructured qualitative interviews and concluded that nonadherence to treatment can be imputed to perception of the patient's doctor. If limited, failure to seek medical counseling was noted [37]. Moreover, in a study carried out by Earnest describing and explaining the patterns of adherence to supplemental oxygen in patients with hypoxemic COPD, it was found that the majority of individuals had a single conversation with their doctors about oxygen that occurred at the time of their initial prescription [35]. The absence of clinician-patient communication at regular intervals may compromise LTOT compliance.

The lack of substantial benefits of treatment from the patients' standpoint might be another reason for the decreased duration of oxygen utilization soon after its initiation. Physicians looking after patients under LTOT quite often hear the following statement: “I breathe oxygen, but I do not see any improvement.” Patients mostly anticipate an immediate positive effect of oxygen therapy confirmed by the improved exercise tolerance. Since the patients do not exhibit that, they feel frustrated and less enthusiastic about continuing the treatment [32].

Disease severity can adversely affect LTOT adherence. COPD in its late stages is a debilitating disease leading to notably poor quality of life. Jones reported that poor functional status for patients is related to depression and feelings of minor support which may translate to inadequate compliance [38]. Symptom management, mainly dyspnea might affect adherence, according to Earnest's study findings [35]. Several participants could tell little difference in how they felt whether they were using oxygen or not. This group of patients, who felt little but immediate benefit in symptom alleviation, struggled more with the role of oxygen in their lives. 

Additionally, poor adherence to LTOT prescriptions may result from associated mental confusion or misunderstanding of the correct prescription. COPD patients with low educational level may be noncompliant with LTOT. Illiteracy to incomprehensible written medical instructions containing scientific terms may lead to inadequate oxygen usage. Accordingly, prescribing physicians should simplify their instructions on LTOT in illiterate patients. Moreover, old age, portable oxygen systems to mobile patients, high PaO_2_ values on room air, and smoking habits comprise potential factors with negative influence on LTOT compliance [39, 40]. COPD hypoxaemic patients, who are active smokers, prefer keeping on smoking to using advisable oxygen therapy resulting in detrimental health consequences.

To recap, Marinker mentioned three rationales that typically explain general nonadherence behavior to medications, given that LTOT is a controller medication [41]. First, patients fear developing immunity if the medication is used for a long period. Second, manufactured materials are not natural; therefore they are not used and thirdly, addiction or dependence may result, or tolerance will develop to the beneficial effects of the medication.

## 5. Methods for Enhancing Adherence to LTOT

Despite the effectiveness of LTOT, as it has been confirmed by the apparent relationship between daily duration of oxygen use (>15 h/d) and survival, adherence remains poor. Therefore, it is incumbent on the clinicians to make strenuous efforts to improve adherence to therapy among their patients who receive oxygen. In this section, a comprehensive report will be performed on the existing literature with regard to strategies that may ameliorate LTOT compliance.

Current methods used to determine LTOT adherence probably overestimate actual use. Each mode of oxygen source utilized for LTOT requires a different way of objective adherence assessment. The daily use of the concentrator is calculated using a time counter attached to the device synchronized with its functional status. For oxygen compressed cylinders, adherence is estimated by knowing the prescribed flow rate and the number of cylinders used. For liquid oxygen, adherence is measured by weighing the container, knowing the flow rate, and estimating the amount of evaporation and venting from the system. But the aforementioned calculations are deficient and probably misleading, since (a) they cannot distinguish whether or not the patient is really inhaling oxygen, as opposed to merely having the device switched on (e.g., oxygen consumption may not reflect oxygen delivery to the patient), and (b) they do not provide precise information concerning the timing of daily oxygen use. Lin et al. recently developed a novel oxygen adherence monitor that objectively measures patients' oxygen use [42]. The monitor attaches to the oxygen delivery system and detects pressure in the tubing, including the respiratory-related pressure fluctuations transmitted from the nares. It was designed to detect when the oxygen source is turned on and when the patient is indeed wearing the nasal cannula and receiving treatment. Lin et al. demonstrated that the monitor was most precise in detecting patients not wearing cannula thus yielding 100% specificity. The most challenging recording situation was noted during sleep, and results showed that accuracy may be decreased in patients with mouth breathing pattern and frequent apneas/hypopneas. However, the monitor's ability to objectively evaluate oxygen delivery, rather than oxygen expended, may contribute to the improvement of patients' adherence to LTOT. 

Regular followup and sincere clinician-patient communication influence positively on LTOT adherence [20]. A well-organized home care program for COPD patients is the cornerstone of a positive attitude of patients regarding LTOT thus improving its efficacy [23, 43]. It is often not appreciated that oxygen is a drug and should therefore be prescribed with due care. Inappropriate LTOT prescriptions can significantly limit patients' independence and in some cases can cause significant morbidity as well as might lead to unjustifiably escalating oxygen costs [44]. Hence, written prescription and precise instructions given by the treating physician regarding LTOT use are indispensable factors affecting adherence. More education is essentially needed in LTOT assessment and management. Due to significance of frequent followup, oximetry is necessary to confirm the necessity and the implementation of prescription. Zhu et al. reported that continuous oximetry reduced liter flow and enhanced use at the desired level [45]. 

Furthermore, the true relationship and communication between physician and patient is instrumental in LTOT compliance. Clinicians involved in LTOT need to be aware and work with the patients to facilitate their use of oxygen. They should conduct a return demonstration and ask the patient to repeat instructions. Oxygen devices are complex and require multiple tasks. Return demonstration of the tasks reflects patient capability. Physicians must discuss with their patients LTOT use on an ongoing basis. More specifically, erroneous beliefs about oxygen use, such as fear of addiction and dependence, stigma, isolation, and embarrassment can be tackled easily through patient-doctor conversation [14, 35, 37, 46]. 

Formal training about appropriate oxygen use has been suggested as an important strategy for adherence improvement. In one study, questionnaires regarding patients' understanding about oxygen usage, their disease process, oxygen safety, and smoking status were administered after six months of education [47]. In the intervention group, 82% were using their concentrator greater than 15 hours per day as compared to 44% of the control group. Accordingly, 93% of intervention patients understood the purpose and hazards of oxygen, while this was true for only 41% of the control group. Interestingly, smoking occurred with one patient in the intervention group and six patients in the control group. 

Family training and social support have also been identified as considerable factors affecting positively compliance. In their study, Ring and Danielson reported that social reinforcement is necessary to ensure understanding for the complexity of the oxygen regimen [48]. Cornford suggested strategies which have been adopted by patients on domiciliary oxygen to maintain control and mastery over their daily lives [34]. For seven of 24 patients using oxygen over 15 h/day, it was used to maintain feelings of independence through relief of breathlessness during daily activities, individual experimentation with the best ways to use it, and reassurance that it was available to be used if necessary.

As to oxygen delivery systems, equipment convenience has been demonstrated as another key factor to adherence improvement [36]. Individuals extremely limited by the weight and bulk of compressed gas in a tank could be switched to concentrators or liquid systems at less than half the weight. Recent studies showed that patients under concentrators complained of machinery noise and increased costs of electricity whereas the absence of noise and the lack of dependence on electricity observed in stationary liquid oxygen users might exert a beneficial effect on patient compliance to LTOT [32, 49]. Other studies found increased duration of oxygen therapy when LTOT recipients were employing portable oxygen sources [28]. 

A stationary oxygen concentrator or liquid oxygen with incorporated tubing up to 50 feet in length, in conjunction with an additional small M-6 cylinder (2 kg, 4 h use) or a small portable liquid reservoir (*∼*2 kg, 5 h use) is an ideal and complete home oxygen system. Portable oxygen concentrators might be an alternative to compressed or liquid oxygen portable systems. They can be powered by house current, an automobile's electrical system, or by a self-contained battery. Their two main disadvantages are the weight (*∼*5 kgs) and their short duration (1–3 hours) on their own batteries. To be out and about for five or more hours would require additional fully charged batteries to replace those as they become fully discharged. This equipment combination provides uninterrupted LTOT since it facilitates patient movements within the home or out into the community [50]. 

However, a major drawback of both of the traditional continuous flow LTOT portable systems is the limited amount of time the patients could be away from their stationary system. The introduction of oxygen-conserving technology offered an innovative solution [51]. Rather than delivering oxygen continuously, an oxygen-conserving device (OCD) dispenses oxygen only intermittently, and particularly a preset volume or bolus of oxygen in response to the patient's inspiratory effort (or demand), as detected through the nasal cannula. OCDs allow patients to spend considerably more time away from the stationary system than continuous flow. Nevertheless, the actual length of time a particular cylinder or liquid oxygen canister will last for with an OCD depends on cylinder/canister size and capacity, the model of OCD being used, and the patient's respiratory rate.

Although traditional portable LTOT systems employing OCD technology have been used successfully for the past 3 decades, one important matter remains: the amount of time a patient may be away from his stationary system is limited. Since one of the biggest fears LTOT patients have is “oxygen depletion,” ambulation is somewhat restricted with traditional LTOT systems. Patients are mindful of returning home to their stationary system before their portable source is totally used up. There are also issues associated with the ongoing need to contact the home-care provider to schedule home deliveries to provide the required refills, and the anxiety of not knowing if the delivery will take place in time for their next outing. On the other hand, oxygen suppliers are equally dealing with their own uncertainties. Since one of the major uncompensated costs that providers encounter is repetitive home deliveries to replenish depleted contents, there is growing concern that the frequency of such deliveries might be further curtailed. Moreover, there is accumulating evidence that patients with COPD who perform a relatively high level of physical activity in their daily life on a sustained basis have a considerably reduced risk of readmission due to an exacerbation [52]. Further, the scientific evidence of the clinical, psychological, and economic advantages of a formal pulmonary rehabilitation program in COPD patients continues to grow [53]. For many COPD patients, successful participation in a pulmonary rehabilitation program entails the need for ample portable oxygen, especially when structured walking exercise is attempted [54]. Any disruption to the unencumbered access to unlimited portable oxygen quickly becomes a disincentive to subsequent participation. 

The aforementioned limitations can be overcome by using newer “nondelivery” oxygen devices. This terminology is due to the fact that the home care provider no longer has to make periodic home deliveries to replenish depleted gaseous or liquid-oxygen contents [55]. A nondelivery LTOT device is self-sufficient and able to provide all of the oxygen needed to meet both stationary and ambulatory requirements. It can be considered a very cost-effective alternative to the expenditures of maintaining traditional stationary and portable systems with repeat home deliveries. 

**Figure 1:**
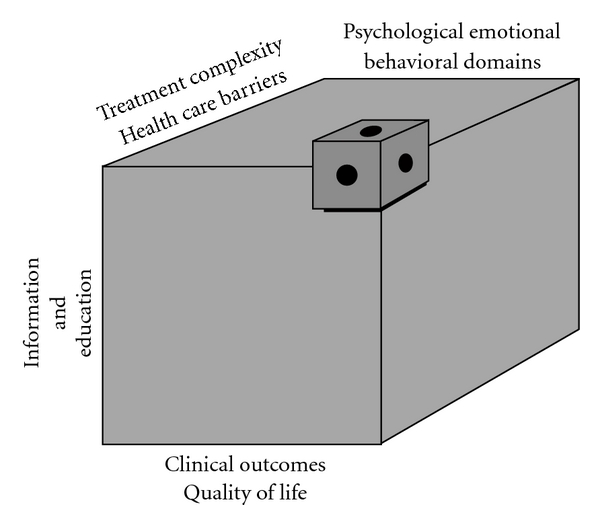
Long-term oxygen therapy: a research agenda for compliance.

As mentioned earlier, studies addressing LTOT have been primarily descriptive and frequently have not included behavioral or psychological approaches. Although some qualitative research has hitherto been performed on LTOT adherence, research focused on the patient's perspective should be expanded [45]. Cullen recently devised a practical research agenda pertinent to identifying research necessary for improving adherence to LTOT (Figure 1).

This research rubric points to four domains for exploration; treatment complexity and health care barriers, aspects related to information and education, and psychosocial, emotional, and behavioral domains. When investigated against clinical outcome measures and quality of life indicators, these broad categories compose a research agenda. Elaborating the research agenda may advance clinical outcomes, quality of life, and self-management knowledge. Only then can comprehensive guidelines be constructed to assist with oxygen self-management.

## 6. Conclusion

It is well substantiated that suboptimal adherence with long-term oxygen therapy is common and causes significant morbidity as well as great expenses to healthcare systems universally. When prescribing medication, it may be important to consider the complexity of the regimen in addition to the efficacy of the intervention. Oxygen is a controller medication, according to the international pharmacopoeia grade, and should be dispensed only upon the written order of a licensed physician. Inappropriate use of oxygen and oxygen-delivery equipment has the potential to result in real harm or injury to the public and cause economic implications. Clinicians involved in LTOT need to be aware and work with the patients to facilitate their use of oxygen. Improving adherence to oxygen therapy and minimizing the negative impacts of the therapy require understanding the subjective experience of the therapy. Exploring patients' concerns and prejudices about oxygen therapy can assist with the development of new interventions and management strategies. Moreover, worthwhile goals for future research include the development of better strategies for patient education and more tolerable methods for oxygen delivery (e.g., oxygen-conserving systems, nondelivery long-term oxygen systems), together with testing of these approaches to verify their effectiveness in improving the outcomes of patients receiving LTOT.

## Figures and Tables

**Table 1:** Overview of adherence evaluation for LTOT.

Source	Subjects/methods	Adherence outcome percent or *M* (SD)	Comments future research needs
Evans et al. [25]	14 Concentrator patients Evaluated usage in comparison to prescribed 15 hours/day	*M* = 13.3 (2) h/d	Patient home respiratory support needed
Vergeret et al. [27]	159 Hypoxic COPD patients Two randomly assigned groups to fixed unit only or fixed plus portable unit	Fixed: *M* = 14 (3) h/d Portable: *M* = 17 (3.5) h/d	Oxygen use and quality of life increased with portable use. Equipment aesthetics and supervision during the first three months needed
Walshaw et al. [21]	61 patients reassessed for use and prescription appropriateness	45.9% inadequate prescription 29.5% compliance with correct prescription	Clinician and patient education should be enhanced
Howard et al. [20]	531 concentrators after use Compared prescription and concentrator clocks	Prescription <15 h/d then Actual use *M* = 9.9 h/d Prescription >15 h/d then Actual use *M* = 13.4 h/d	LTOT is complex and education for rationale disease management needed. Regular home care is necessary
Restrick et al. [29]	176 patients interviewed and followed up	74% used 12 + h/d	Reassessment necessary. Greater communication among providers
Morrison and Stovall [10]	630 LTOT patients 79% were COPD Database evaluation for three years	*M* = 14.9 h/d 44% was less than 15 h/d	Instruction needed at time of prescription but also followup later when clinically stable
Pépin et al. [19]	930 COPD patients Compared prescription and actual use	45% achieved 15 + h/d Prescription *M* = 16 h/d Actual use *M* = 14.5 h/d	Education at prescription needed and more prospective educational intervention studies necessary
Granados et al. [22]	62 LTOT patients participated 70% were COPD Evaluated compliance and if hypoxemia was corrected	31% met all criteria for adherence to adequate prescription 61% measured as compliant	Therapeutic process is noted as prescription, oxygen device, and compliance. Chronic care requires reassessment
Ringbæk et al. [28]	125 of 182 LTOT patients surveyed and evaluated as to activities and portable oxygen use	65% acceptable compliance Ambulatory use positively affected compliance	Need to discuss how and when LTOT is used and portable oxygen options needed
Atiş et al. [30]	379 of 1100 patients responded to questionnaire	28.2% self-reported use was 15 + h/d *M* = 9 h/d	Physician instruction and followup produced greater use by patients
Katsenos et al. [23]	249 LTOT patients 75% were COPD patients	26.9% complied. 55% did not receive precise written instructions concerning LTOT. 63% did not know LTOT importance for the management of COPD	A well-organized home care program may check LTOT utility and enhance its efficacy in COPD patients
Lacasse et al. [31]	24 hypoxic COPD patients were allocated to three interventions: oxygen concentrator only, concentrator plus as-needed ambulatory oxygen and concentrator plus ambulatory compressed air. Comparison of home-based oxygen therapy alone with ambulatory oxygen added to home-based oxygen	Concentrator use: 18 h/day Concentrator plus ambulatory oxygen: 17.4 h/day Concentrator plus ambulatory compressed air: 18 h/day	The widespread provision of portable oxygen-dependent COPD patients is not justified. The efficient use of ambulatory oxygen in a successful course of respiratory rehabilitation remains to be determined
Nasiłowski et al. [32]	30 patients under LTOT (77% COPD patients) were followed up for14 consecutive months	37% compliance. Higher compliance (48%) during the first month. Nurses' frequent home visits did not increase compliance. Noise produced by concentrator influenced significantly the compliance	An alternative oxygen source, which would not generate any noise or electricity consumption may positively affect the compliance

**Table 2:** Factors influential to patient adherence.

Illness factors	Personal/family factors
Illness characteristics	Demographic factors
Treatment complexity	Patient/Family functioning
Attitudes toward LTOT	Cognitive factors (e.g., health literacy)
